# Trend of serum C-reactive protein is associated with treatment outcome of hip Periprosthetic joint infection undergoing two-stage exchange arthroplasty: a case control study

**DOI:** 10.1186/s12891-021-04893-3

**Published:** 2021-12-02

**Authors:** Zhong-Yan Li, Yu-Chih Lin, Chih-Hsiang Chang, Szu-Yuan Chen, Tung-Wu Lu, Sheng-Hsun Lee

**Affiliations:** 1 Department of Medicine, Chang Gung University, No.259, Wenhua 1st Rd., Guishan Dist., Taoyuan, 33302 Taiwan, Republic of China; 2 Department of Orthopaedic Surgery, Chang Gung Memorial Hospital, Linkou, No.5, Fuxing St., Guishan Dist., Taoyuan, 33305 Taiwan, Republic of China; 3 Bone and Joint Research Center, Chang Gung Memorial Hospital, Linkou, No.5, Fuxing St., Guishan Dist., Taoyuan, 33305 Taiwan, Republic of China; 4 Department of Biomedical Engineering, National Taiwan University, No. 1, Sec. 4, Roosevelt Rd., Taipei, 10617 Taiwan, Republic of China

**Keywords:** Two-stage exchange arthroplasty, Periprosthetic joint infection, CRP trends, Spacer-related complications

## Abstract

**Background:**

Serum C-reactive protein (CRP) trends are critical for monitoring patients’ treatment response following a two-stage exchange arthroplasty for periprosthetic joint infection (PJI) of the hip. However, CRP trends are poorly described in the literature. The primary aim of this study was to identify the relationships between PJI treatment outcomes and our proposed CRP trend definitions, parameters, and microbiological data. The secondary aim was to investigate CRP trends after the occurrence of spacer-related complications.

**Methods:**

We conducted a retrospective review of 74 patients treated with a two-stage exchange protocol for PJI in a tertiary referral joint center between 2014 and 2016. Patients with factors that may affect CRP levels (inflammatory arthritis, concomitant infections, liver and kidney diseases, and intensive care admissions) were excluded. CRP trends were categorized into five types and PJI treatment outcome was defined as “success” or “failure” according to the Delphi criteria.

**Results:**

Treatment was successful in 67 patients and failed in 7 patients. Multivariate logistic regression analysis showed that type 5 CRP, defined as serum CRP fluctuation without normalization after first stage surgery (odds ratio [OR]: 17.4; 95% confidence interval [CI]: 2.3–129.7; *p* = 0.005), and methicillin-resistant *Staphylococcus aureus* (MRSA; OR: 14.5; 95% CI: 1.6–131.7; *p* = 0.018) were associated with treatment failure. Spacer-related complications occurred in 18 patients. Of these, 12 had elevated CRP levels at later follow-up, while six had no elevation in CRP levels.

**Conclusions:**

We found that MRSA infection and type 5 CRP were associated with PJI treatment failure.

## Introduction

Periprosthetic joint infection (PJI) is a common cause of failure following total hip arthroplasty and imposes a great burden on economic and medical resources [1–3]. Two-stage exchange arthroplasty remains the gold standard for PJI treatment [4]. The treatment consists of a first-stage resection arthroplasty followed by antibiotic therapy with an interim spacer, and finally a second-stage reimplantation.

Diagnostic criteria of PJI was established by Musculoskeletal Infection Society (MSIS), including serum C-reactive protein (CRP) [5]. CRP is an acute phase reactant, which increases in infectious diseases and non-infectious inflammatory disorders, such as rheumatoid arthritis, systemic lupus erythematous, kidney or liver diseases [6–8]. With its verified role in PJI diagnosis, serum CRP in the interim period between two stages is highly emphasized but poorly studied. Serum CRP has been used to monitor treatment response, and a downtrend of CRP is considered one of the metrics for reimplantation [9].

Spacers are widely used during the interim period in two-stage exchange arthroplasty and perform two vital roles in the treatment of PJI: 1) they provide an environment with consistently high antibiotic concentrations, and 2) they maintain basic joint functions. Spacer fracture or dislocation may occur prior to reimplantation, however [10–12]. When a patient presents with elevated CRP and signs of a malfunctioning spacer, which causes pain and dysfunction, it may become more difficult for the clinicians to evaluate the infection status based on serum CRP and to determine the more effective treatment strategy (spacer exchange or second-stage reimplantation).

CRP trend has been a subjective observation by clinicians and never been described in an organized way before. One indicator for PJI treatment response is synovial fluid analysis. It is more difficult to obtain synovial fluid in outpatient clinical settings, especially for hip PJI. As a result, other parameters such as serological markers appear to be more crucial.

The primary aim of this study was to propose new definitions for CRP trends, parameters, and microbiological data, and correlate these with PJI treatment outcome after two-stage exchange arthroplasty. The secondary aim was to investigate CRP trends following spacer-related complication events.

## Methods

After obtaining institutional review board approval (102-1846B), we conducted a retrospective review of patients with PJI of the hip who were treated at a tertiary referral joint center between 2014 and 2016.

### Inclusion/exclusion criteria

Patients with chronic hip PJI and received two-stage revision surgery were included. PJI in the current study was defined by the criteria established by MSIS in 2011 and later modified by International Consensus Meeting in 2013 [13]. Two “major criteria” (two positive periprosthetic cultures, sinus tract) and five “minor criteria” (elevated CRP and erythrocyte sedimentation rate [ESR] levels, elevated synovial fluid white blood cell count or ++ change on leukocyte esterase test strip, elevated synovial fluid polymorphonuclear neutrophil percentage, positive histological analysis of periprosthetic tissue, a single positive culture) were incorporated. PJI was diagnosed if either one of the major criteria or three of the minor criteria were met.

Patients with PJI occurring within 3 months of index surgery, pre-existing hardware from fracture fixation, or additional debridement within 4 weeks after resection were excluded. Patients were excluded if they had any of the following confounding factors (conditions that may also affect serum CRP): inflammatory arthritis (rheumatoid arthritis, systemic lupus erythematous, etc.), liver cirrhosis, hepatitis, chronic kidney disease, concomitant infection (PJI in another joint, urinary tract infection, pneumonia), or admission to an intensive care unit. Patients with spacer-related complications, including spacer fracture and dislocation, which may adversely affect serum CRP trends, were excluded from the final analysis of the correlation between CRP trends and PJI treatment outcomes.

### Patient demographics

The primary study excluded 24 patients who had CRP confounding factors and 18 patients with spacer-related complications, leaving 74 patients who were eligible for the final analysis. All patients had more than 2 years of follow-up and completed two-stage exchange arthroplasty. Of these, 68 patients received reimplantation without an interim spacer, while the remaining six received additional debridement and spacer exchange, followed by reimplantation.

The following patient information was recorded: age, sex, body mass index (BMI), Charlson Comorbidity Index (CCI), presence of sinus tract, chronic PJI (symptoms lasting > 4 weeks), interim period between first- and second-stage surgery, microbiological data, and surgical parameters of second-stage surgery (operation time and blood loss).

### Treatment protocol

The treatment protocol for chronic hip PJI in our institution is two-stage exchange arthroplasty. During first-stage surgery, we would obtain synovial fluid for routine analysis, and at least 5 periporsthetic cultures, including tissue cultures and synovial fluid culture in blood culture bottle. The prostheses and bone cement were removed, soft tissue and bone debridement were performed meticulously, and the surgical site was irrigated with more than 5 L of normal saline solution. A total-hip type of mobile spacer was then implanted with high-dose antibiotic-loaded polymethylmethacrylate (PMMA). The spacer was impregnated with an antibiotic combination of 4 g vancomycin and 4 g ceftazidime per 40 g PMMA powder. According to previous in vitro studies, these antibiotic-loaded spacers can provide sufficient mechanical strength and antibiotic elution and concentration profiles for effective eradication of PJI [14–17].

Organism-specific intravenous (IV) antibiotics were administered 2 weeks after first-stage surgery, followed by 4 weeks of oral antibiotics in the interim period [14, 18, 19]. Infectious disease specialists would only be consulted for antibiotic recommendations if the pathogen could not be identified. Patients were followed up 2 weeks after discharge at an orthopedic clinic, then every 2–4 weeks thereafter, as per the surgeon’s treatment protocol. A clinical assessment was conducted, wound condition and serum CRP levels were evaluated at each visit.

Further debridement with spacer exchange would be performed if the patient had experienced persistent wound drainage, sinus discharge, or other indicative signs of persistent infection. Currently there is no definite criteria for the optimal timing of second stage surgery in the literature. Reimplantation was performed only when deemed appropriate by the attending surgeon. Cultures were then obtained before antibiotic administration. All patients other than those who were culture-positive were administered with IV antibiotics within 24 h after reimplantation.

### Evaluation criteria for PJI treatment outcomes

For the evaluation of PJI treatment outcome, there has not been a universal agreement [20]. We adopted two outcome definitions including the international multidisciplinary Delphi consensus (Delphi criteria) and modified Delphi consensus (modified Delphi criteria). Delphi criteria consists of lack of the following conditions: (1) the presence of a sinus tract, an unhealed wound drainage, pain, or infection recurrence caused by the same strain of organism, (2) subsequent surgical intervention for infection after reimplantation surgery, (3) occurrence of PJI-related mortality [21]. In contrast, treatment “failure” according to the modified Delphi criteria differs from the original Delphi criteria in the following ways: (1) the definition of reinfection is not limited to the same organism, and (2) it includes any unplanned surgical intervention, including spacer exchange [20]. For patients underwent debridement in the interim period but were reimplanted and remained infection-free, they were considered treatment success by Delphi criteria, but failure by modified Delphi criteria.

Patients were categorized as treatment “success” or “failure” according to the Delphi criteria (Table 1). Both the Delphi and the modified Delphi criteria were used to identify correlations between CRP trends and treatment outcomes (Table 2). When the modified Delphi criteria were used, trends in CRP were evaluated from the first-stage resection to either the second-stage reimplantation or the spacer exchange to assess treatment outcomes.

**Table 1:** Summary of treatment groups

	Treatment success (***n*** = 67)	Treatment failure (***n*** = 7)	***p***
**Age (years)**^a^	58.6 ± 12.7	49.6 ± 14.5	0.034*
**Female (%)**	26 (38.8)	0 (0)	0.047*
**BMI (kg/m**^**2**^**)**^a^	25.9 (24.0–27.8)	30.1 (28.5–31.7)	0.102
**CCI ≥ 4 (%)**	29 (42.6)	3 (50)	0.995
**Sinus discharge (%)**	16 (23.9)	3 (42.9)	0.363
**Chronic PJI (%)**	44 (65.7)	6 (85.7)	0.416
**Interim period (weeks)**	13.0 (12.0–15.6)	13.0 (10.0–20.0)	0.572
**2nd-stage surgery time (hours)**	2.4 (1.9–3.1)	3.1 (2.4–4.1)	0.102
**2nd-stage blood loss (mL)**	975 (725–1625)	2200 (1000–2300)	0.056
**Pre-resection CRP**^b^	29.9 (14.8–74.1)	41.3 (9.5–102.1)	0.737
**ΔCRP**^b^	22.1 (10.5–62.7)	18.8 (6.1–48.2)	0.747
**ΔCRP/pre-resection CRP**^b^	0.85 (0.63–0.95)	0.64 (0.28–0.84)	0.278
**Pre-reimplantation CRP**^b^	3.6 (1.8–7.3)	20.2 (10.4–33.6)	0.008*
**CRP decrease ratio**^b^			
1st vs. 2nd week	47.7 (35.8–67.1)	42.8 (−18.1–55.7)	0.477
2nd vs. 3rd week	40.0 (15.1–68.1)	27.1 (−2.5–52.1)	0.832
1st vs. 3rd week	75.3 (55.1–85.9)	48.7 (3.9–0.7)	0.323
**Pathogen**			
No growth	24 (35.8)	0 (0)	–
MRSA	4 (5.9)	2 (28.6)	0.046*
MSSA	20 (29.9)	2 (28.6)	0.939
Other G(+)	8 (11.9)	2 (28.6)	0.245
G(−)	5 (7.5)	1 (14.3)	–
Fungus, mycobacteria	3 (4.5)	0 (0)	–
Polymicrobial	3 (4.5)	0 (0)	–

^a^Results are expressed as mean ± standard deviation

^b^Results are expressed as median (interquartile range)

* *p* < 0.05

*Abbreviations*: *BMI* body mass index, *CCI* Charlson Comorbidity Index, *CRP* C-reactive protein, *G(+)* gram positive, *G(−)* gram negative, *MRSA* methicillin-resistant *Staphylococcus aureus*, *MSSA* methicillin-sensitive *Staphylococcus*, *PJI* periprosthetic joint infection

**Table 2:** Correlation of CRP trends and treatment outcomes

CRP type	Modified Delphi criteria					Delphi criteria				
	Success (*n* = 63)	Failure (*n* = 11)	OR	95% CI	*p*	Success (*n* = 67)	Failure (*n* = 7)	OR	95% CI	*p*
**1**	33	0	–		–	33	0	–		–
**2**	13	1	–		–	13	1	–		–
**3**	8	1	–		–	8	1	–		–
**4**	5	2	10.8	1.2–93.9	0.031*	9	2	–		–
**5**	4	7	47.3	7.3–306.9	0.001*	5	3	9.1	1.6–53.7	0.004*

* *p* < 0.05, type 1 as reference

*Abbreviations*: *CI* confidence interval, *CRP* C-reactive protein, *OR* odds ratio

We developed the following definitions of CRP trends, categorized into five types, to help identify any relationships between serum CRP trends and treatment outcomes:

- Type 1: CRP drops below the threshold value of 10 mg/L within 3 weeks after resection.
- Type 2: CRP drops below 10 mg/L beyond 3 weeks after resection, with a single CRP outlier above 10 mg/L allowed.
- Type 3: CRP is consistently below 10 mg/L before and after resection.
- Type 4: CRP fluctuates after resection, but drops below 10 mg/L at least once.
- Type 5: CRP fluctuates after resection, but remains above 10 mg/L.

Several CRP parameters were defined, including pre-resection CRP, pre-reimplantation CRP, ΔCRP (change in CRP levels between pre-resection and pre-reimplantation), and ΔCRP/pre-resection CRP ratio.

### Statistical analysis

All parametric numerical data are expressed as mean ± standard deviation, nonparametric numerical data as median and interquartile range (IQR), and categorical data as numbers and percentages.

Mann-Whitney *U* tests and Fisher’s exact tests were used for numerical and categorical comparisons, respectively. Univariate logistic regression was used for microbiological analysis. Multivariate logistic regression, incorporating stepwise selection, was performed to assess univariate factors, which were either statistically significant or clinically important. Kaplan–Meier survival analysis and log-rank tests, with endpoints defined based on the Delphi criteria, were used to compare the survival outcomes of patients with types 1–4 CRP and type 5 CRP. All tests were two-sided, with *p* < 0.05 indicating statistical significance. Statistical analyses were performed using MedCalc Statistical Software version 19.2.6 (MedCalc Software, Ostend, Belgium).

## Results

### Overall patient demographics

There were 67 patients in the treatment success group and seven patients in the treatment failure group according to the Delphi criteria. The treatment success group comprised 63 patients who received second-stage reimplantation without an additional surgery in interim period and four who received a spacer exchange followed by reimplantation.

The median follow-up period for the treatment success group was 211.2 weeks (IQR: 181.8–265.6 weeks), while the median time-to-failure for the treatment failure group was 34.7 weeks (IQR: 3.8–70.2 weeks). Detailed comparisons of the treatment groups are provided in Table 1**.** The treatment success group was significantly older (58.6 years vs. 49.6 years) and had a higher proportion of females (26 patients vs. 0 patients) than the treatment failure group.

### Primary outcomes

**Fig. 1:**
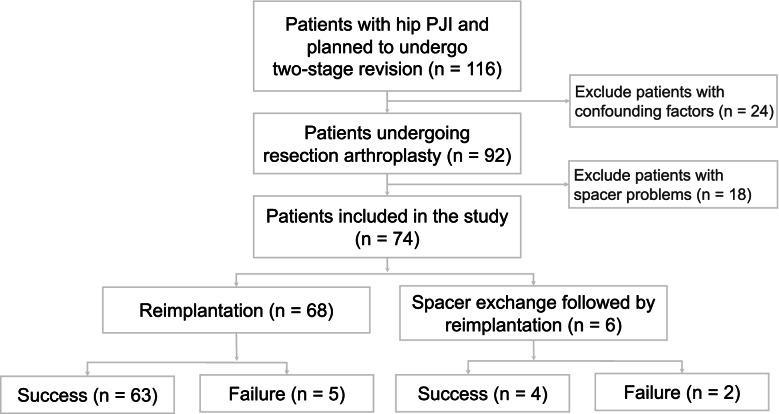
Patient inclusion flowchart. The criteria for treatment success and failure were defined based on the Delphi criteria

Sixty-eight of the patients underwent two-stage surgery without interim debridement (Fig. 1), five of whom were in the treatment failure group, according to the Delphi criteria. Of these, one patient with type 4 CRP had positive intraoperative cultures during the second-stage surgery, and the remaining four patients (who had type 2, 3, 4, and 5 CRP, respectively) underwent debridement 2–32 weeks after reimplantation.

Debridement and spacer exchange after first-stage surgery were performed in six patients. Treatment outcomes were correlated to the CRP trends: (1) the four patients in the treatment success group had a type 5 CRP that changed to type 4 after the debridement; and (2) the two patients in the treatment failure group had a type 5 CRP that did not change after the debridement.

Pre-reimplantation CRP was higher in treatment failure group (3.6 versus 20.2, *p* = 0.008). There were no significant differences between pre-resection CRP, ΔCRP, or the ΔCRP/pre-resection CRP ratio between treatment outcome groups. There were also no significant decreases in the ΔCRP/pre-resection CRP ratio among the three observed time intervals: first vs. second week, second vs. third week, and first vs. third week.

There were six patients who were infected with methicillin-resistant *Staphylococcus* (MRSA), 22 with the nonresistant strain of *Staphylococcus*, 10 with other gram-positive bacteria, six with gram-negative bacteria, two with fungus, one with mycobacteria, and three who had polymicrobial infections. MRSA was significantly associated with treatment failure (odds ratio [OR]: 5.44; *p* = 0.046).

Most of the patients with types 1–3 CRP were in the treatment success group (Table 2), and patients with type 5 CRP had the lowest success rate. Based on the modified Delphi criteria, patients with type 4 (OR: 10.8; 95% confidence interval [CI]: 1.2–93.9; *p* = 0.031) and type 5 CRP (OR: 47.3; 95% CI: 7.3–306.9; *p* = 0.001) had significantly higher odds of treatment failure than those with type 1 CRP. Similarly, based on only the Delphi criteria, type 5 CRP was associated with treatment failure, with an OR of 9.1 (95% CI: 1.6–53.7; *p* = 0.004).

The multivariate analysis identified type 5 CRP (OR: 17.4; 95% CI: 2.3–129.7; *p* = 0.005) and MRSA (OR: 14.5; 95% CI: 1.6–131.7; *p* = 0.018) as two major risk factors for treatment failure (Table 3). The *p* value of the overall model fit was 0.002. Age, sex, BMI, all pathogens except MRSA, and all CRP types except for type 5 were excluded from the final model.

**Table 3:** Major risk factors for treatment failure

Independent variable	Coefficient	Standard error	OR	95% CI	*p*
**CRP type 5**	2.86	1.02	17.4	2.3–129.7	0.005*
**MRSA**	2.67	1.26	14.5	1.6–131.7	0.018*

* *p* < 0.05

*Abbreviations*: *CI* confidence interval, *CRP* C-reactive protein, *MRSA* methicillin-resistant *Staphylococcus aureus*, *OR* odds ratio

**Fig. 2:**
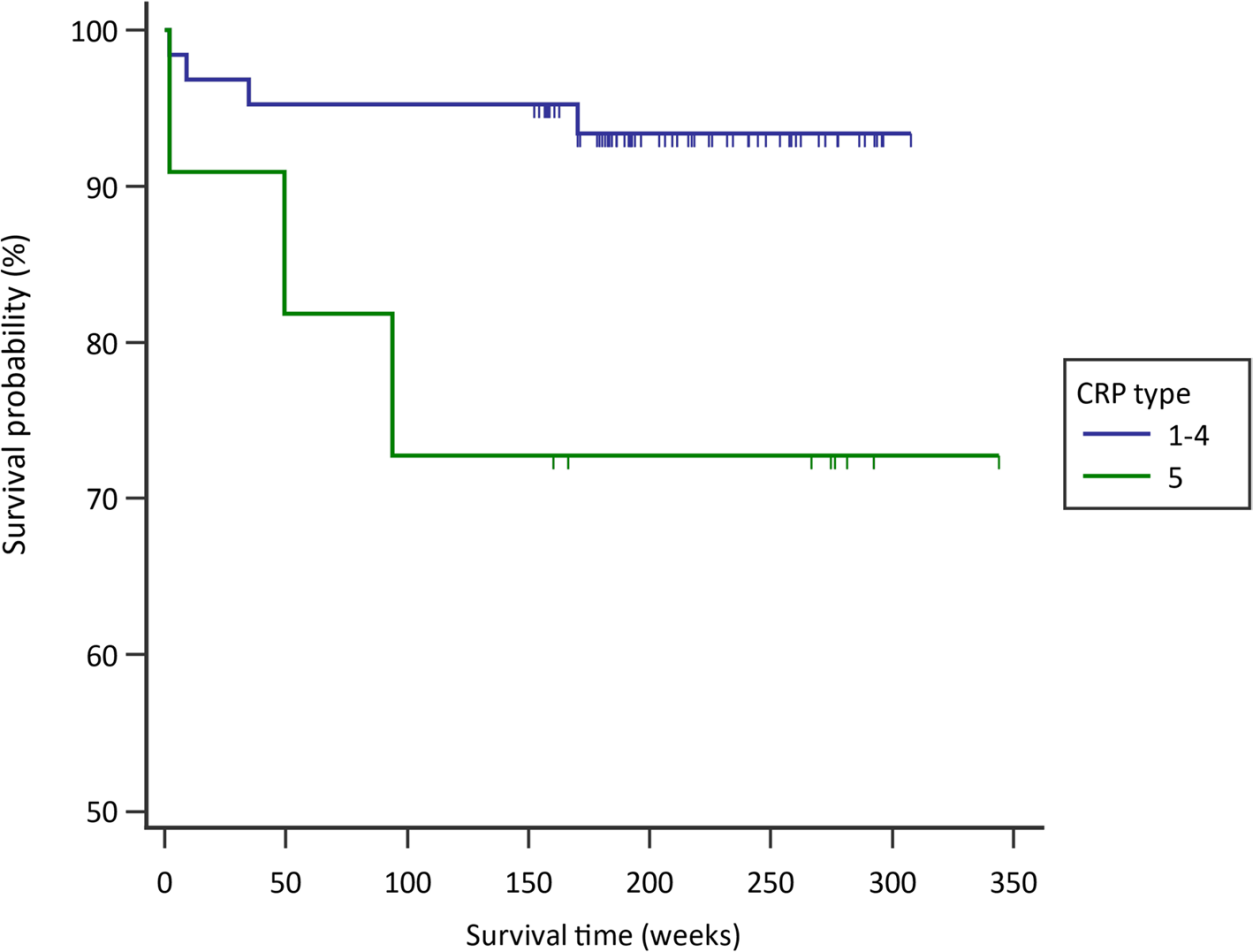
Kaplan–Meier survival curves for patients with types 1–4 CRP (blue line) and type 5 CRP (green line). The difference between two groups was significant (*p* = 0.025 with Logrank test)

Kaplan–Meier survival analysis revealed significant differences between patients with types 1–4 CRP and type 5 CRP (Fig. 2). The log-rank test also revealed significant differences between these two groups (*p* = 0.021).

### Secondary outcomes

**Fig. 3:**
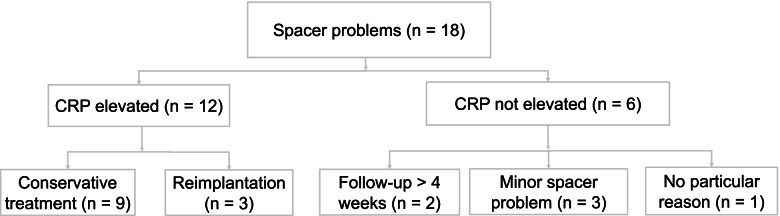
Flowchart of patients with spacer-related complications

Spacer-related complications were observed in 18 patients on plain radiographs (Fig. 3). Elevated levels of serum CRP were reported in 12 of these patients. Of these, three continued with reimplantation within 1 week of occurrence, because the surgeons deemed them “infection-free”, while the other nine were treated conservatively and exhibited reduced serum CRP levels at the two-week follow-up (median decline in CRP: 64.4%; IQR: 38.1–90.6%).

CRP elevations were not detected in six of these patients, and most of these observations had possible explanations: two patients had a four-week delayed serum CRP analysis after spacer-related complications were detected, and three patients had minor spacer-related complications (two with crack at the spacer neck without deformations and one patient with minor spacer subsidence). All 18 patients were reimplanted successfully without infection recurrence.

## Discussion

The International Consensus on Orthopedic Infections and numerous studies have described CRP trend as a critical parameter for assessing PJI treatment response after the first-stage resection in a two-stage exchange arthroplasty [22–26]. However, there is still a knowledge gap in terms of clear definitions of CRP trends and their relationship with PJI treatment outcomes. Our study has found that PJI treatment failure is correlated with type 5 CRP, which we have defined as fluctuations in CRP levels after the first-stage resection, but with no observed drop below the threshold value of 10 mg/L. It was interesting that those patients in the treatment success group who underwent additional debridement in the interim period exhibited a change in their CRP trend from type 5 to type 4 after debridement. These observations may imply a high possibility of treatment success if the CRP level drops below the threshold value of 10 mg/L at least once during the interim period. Patients with CRP type 1, namely that CRP dropped below 10 mg/L within 3 weeks after resection, were of high probability of treatment success. In addition, patients whose CRP dropped below 10 mg/L but at a slower rate (type 2), or CRP was consistently low (type 3), there was a good chance of treatment success.

In this study we have reported CRP trends that can provide a timely indication of infection status. CRP levels that do not decline to normal levels during the interim period may indicate that the current treatment protocol has a lower chance of achieving successful infection control. However, CRP levels that normalize at least once during the interim period may suggest that a successful PJI treatment is likely, although the presence of a low-grade infection or other underlying factors may prevent the CRP levels from consistently falling below 10 mg/L during this period.

The timing of reimplantation is still controversial and mostly depends on clinical judgement and laboratory data, including subjective patient complaints, wound and soft tissue condition, serum inflammatory markers, and synovial fluid analysis. Although the synovial fluid white blood cell count can effectively predict the infection status of the hip, this parameter is difficult to obtain in most outpatient clinical settings. Therefore, serum inflammatory markers such as ESR and CRP are widely used to monitor treatment response. At the institution where this study was carried out, CRP (an acute-phase reactant) is more frequently used than ESR, which is a marker for chronic inflammation [21]. Previous studies have examined the relationship between pre-reimplantation serum CRP and PJI treatment outcome. Shukla et al. found above-normal pre-reimplantation CRP in 25% of their patients in the treatment success group [24], whereas other authors have found no correlation between pre-reimplantation CRP and treatment outcome [23, 25–28]. In the current study we recorded a median pre-reimplantation CRP of 3.6, with an IQR of 1.8–7.3, for the treatment success group. The differences in CRP levels between our study group and that of Shukla et al. could arguably be attributed to the shorter interim-period interval (mean 74.5 days) reported by the latter, hence there was less time for CRP to normalize despite the infection had been eradicated. In addition, we reported significantly higher median pre-reimplantation CRP levels for the treatment failure group (20.2 mg/L vs. 3.6 mg/L). With that being said, we argue that CRP value at a given time point is too sensitive to any stress, including persistent PJI, minor infection in another organ, or discomfort from the joint. As a result, a single CRP value might not be a suitable parameter to predict treatment outcome.

Stambough et al. reported that ΔCRP/preresection CRP was not associated with reinfection risk [29]. In the current study, slightly higher ΔCRP/preresection CRP ratio was observed in treatment success group, though not statistically significant (*p* = 0.278). We also investigated the rate of CRP decrease within 3 weeks after resection. There was a trend that CRP decreased more rapidly in treatment success group, but still not reached statistical significance.

Another commonly used serum biomarker is ESR. Dwyer et al. reported higher pre-resection ESR in patients with recurrent PJI [30]. However, due to high variability within their study population and treatment durations (30 surgeons at four institutions, spanning 15 years) and, most importantly, exclusion criteria that would obscure ESR values, their results may not be convincing. Maier et al. found a higher presurgery ESR/CRP ratio in their treatment failure group for patients with chronic PJI undergoing debridement, antibiotics, and implant retention (DAIR) [31]. It is reasonable to assume that DAIR, which is more appropriate for acute PJI because of the greater development of time-dependent biofilms as a result of longer infection times, would lead to higher ESR values and failure rates. The current study did not analyze the usefulness of ESR because only one third of the patients had pre-resection ESR value, and biofilm is a less important issue when the two-stage protocol was utilized.

MRSA has been reported to be a risk factor for PJI treatment failure [27, 32]. After adjusting for confounding factors, we identified MRSA as a risk factor that may have increased the probability of treatment failure in the current study (*p* = 0.020). This finding highlighted the need for more meticulous follow-up and possibly different treatment protocols for patients with this risk factor.

Variations in the definitions of treatment outcomes greatly influence “treatment success” rates and PJI-associated research results [20]. Our study corroborates the research of Tan et al. and proposes a clear definition for PJI treatment outcomes. Further, we report treatment success rates of 85 and 92% based on the modified Delphi and Delphi criteria, respectively, both of which are comparable to previous studies [14, 19]. The major differences of the two definitions include debridement during the interim period, a positive culture of various organisms, and the cause of mortality.

A CRP threshold value of 10 mg/L had been proposed by Ghanem et al. and was later adopted by MSIS in their treatment criteria [33, 34]. A previous study reported a peak in CRP values 3 days after primary total knee arthroplasty (TKA), with a return to normal levels at 2 weeks [35]. Another study reported a peak in CRP values at 2 days, followed by a decline to a median of 12 mg/L at week 2, but no return to baseline [36]. Here we have proposed a reasonable cutoff of 3 weeks for type 1 and 2 CRP to explore the relationship between CRP decline rate and treatment outcomes.

A previous study whose cohort of patients had low CRP values throughout a two-stage treatment course reported PJI with a low-virulence microorganisms, yielding lower CRP levels [34]. However, we did not have similar observations. In our study, there were two patients with MRSA infection, one with methicillin-sensitive *Staphylococcus* (MSSA), one with *Candida*, one with *Propionibacterium*, and four with culture-negative results in type 3 CRP pattern.

Spacer-related complications such as dislocation and spacer fracture have been reported in 10–40% of patients treated with the two-stage exchange protocol [10–12]. In the current study there were 18 patients (19.6%) who had spacer-related problems, including three patients with minor spacer complications (spacer cracks without deformation and mild spacer subsidence). Increased CRP levels were detected in two-thirds of these patients. CRP levels decreased significantly in all of these patients after 2 weeks (range: 38.07–90.59%), and they all remained infection free. To our knowledge, this is the first study to highlight a relationship between spacer-related complications and elevated CRP. When we encounter patients with elevated CRP as well as spacer problems, one reasonable measure is wait and see until next follow-up, because oftentimes the CRP is masked by the discomfort induced by spacer problems.

This study had several strengths. Firstly, we did not solely focus on a single CRP level at a given time point, but instead observed serial CRP trends over the interim period. Secondly, confounding factors that would obscure changes in and affect CRP levels, such as patients with inflammatory arthritis, concurrent infection of other organs, or renal or liver diseases, were excluded from the final analysis. The study also had some limitations, however. Firstly, this was a retrospective review of patients from a certain period and different surgeons, which may have resulted in gaps in demographic data in the two treatment groups and disparities in the CRP follow-up protocols. Secondly, our classification of CRP trends was somewhat arbitrary. However, this may have helped to describe trends while minimizing outlier values. Thirdly, despite assays of more than five cultures during the first-stage resection, there were 32.4% of patients with culture-negative results, which may suggest that the microorganism analysis was somewhat unconvincing. This phenomenon may have been influenced by the large percentage of patients who had been referred to the institution following failed treatment at other hospitals.

## Conclusions

This study showed that hip PJI patients with MRSA infection or type 5 CRP, defined as fluctuations in CRP levels that never dropped below the 10 mg/L threshold value, were associated with treatment failure after two-stage exchange arthroplasty. This finding highlights the importance of healthcare providers meticulously following-up patients with these risk factors and considering alternative treatment protocols, such as more aggressive surgical debridement and a longer duration of antibiotic treatment.

## Data Availability

The datasets used during the current study are available from the corresponding author on reasonable request.

## Abbreviations

BMI: Body mass index
CCI: Charlson comorbidity index
CRP: C-reactive protein
CI: Confidence interval
ESR: Erythrocyte sedimentation rate
IQR: Interquartile range
IV: Intravenous
MRSA: Methicillin-resistant *Staphylococcus aureus*
MSIS: Musculoskeletal Infection Society
MSSA: Methicillin-sensitive *Staphylococcus aureus*
OR: Odds ratio
PMMA: Polymethylmethacrylate
PJI: Periprosthetic joint infection
