# Correction to: Trend of serum C-reactive protein is associated with treatment outcome of hip Periprosthetic joint infection undergoing two-stage exchange arthroplasty: a case control study

**DOI:** 10.1186/s12891-022-05002-8

**Published:** 2022-01-15

**Authors:** Zhong-Yan Li, Yu-Chih Lin, Chih-Hsiang Chang, Szu-Yuan Chen, Tung-Wu Lu, Sheng-Hsun Lee

**Affiliations:** 1 Department of Medicine, Chang Gung University, No.259, Wenhua 1st Rd., Guishan Dist, Taoyuan, 33302 Taiwan Republic of China; 2 Department of Orthopaedic Surgery, Chang Gung Memorial Hospital, Linkou, No.5, Fuxing St., Guishan Dist, Taoyuan, 33305 Taiwan Republic of China; 3 Bone and Joint Research Center, Chang Gung Memorial Hospital, Linkou, No.5, Fuxing St., Guishan Dist, Taoyuan, 33305 Taiwan Republic of China; 4 Department of Biomedical Engineering, National Taiwan University, No. 1, Sec. 4, Roosevelt Rd., Taipei, 10617 Taiwan Republic of China

**Correction to: BMC Musculoskelet Disord 22, 1007 (2021)**

**https://doi.org/10.1186/s12891-021-04893-3**

Following the publication of the original article [1] the authors noticed that the affiliations for authors Zhong-Yan Li, Tung-Wu Lu, and one affiliation of Sheng-Hsun Lee are incorrect.

The affiliations of these authors should be:

**Zhong-Yan Li**:

*Department of Medicine, Chang Gung University, No.259, Wenhua 1st Rd., Guishan Dist., Taoyuan 33302, Taiwan, Republic of China*

**Tung-Wu Lu**:

*Department of Biomedical Engineering, National Taiwan University, No. 1, Sec. 4, Roosevelt Rd., Taipei 10617, Taiwan, Republic of China*

**Sheng-Hsun Lee**:

*Department of Orthopaedic Surgery, Chang Gung Memorial Hospital, Linkou, No.5, Fuxing St., Guishan Dist., Taoyuan 33,305, Taiwan, Republic of China*

*Bone and Joint Research Center, Chang Gung Memorial Hospital, Linkou, No.5, Fuxing St., Guishan Dist., Taoyuan 33305, Taiwan, Republic of China*

*Department of Biomedical Engineering, National Taiwan University, No. 1, Sec. 4, Roosevelt Rd., Taipei 10617, Taiwan, Republic of China*

The original article [1] has been updated.
